# The Associations between Sex Hormones and Lipid Profiles in Serum of Women with Different Phenotypes of Polycystic Ovary Syndrome

**DOI:** 10.3390/jcm10173941

**Published:** 2021-08-31

**Authors:** Anna Bizoń, Grzegorz Franik, Justyna Niepsuj, Marta Czwojdzińska, Marcin Leśniewski, Artur Nowak, Malwina Szynkaruk-Matusiak, Paweł Madej, Agnieszka Piwowar

**Affiliations:** 1 Department of Toxicology, Faculty of Pharmacy, Wroclaw Medical University, 50-556 Wroclaw, Poland; agnieszka.piwowar@umed.wroc.pl; 2 Department of Endocrinological Gynecology, Medical University of Silesia, 40-752 Katowice, Poland; antoni.franik@gmail.com (G.F.); pmadej@sum.edu.pl (P.M.); 3 Students Scientific Society at the Department of Biomedical and Environmental Analyses, Faculty of Pharmacy, Wroclaw Medical University, 50-556 Wroclaw, Poland; justyna.niepsuj@student.umed.wroc.pl; 4 Students Scientific Society at the Department of Toxicology, Faculty of Pharmacy, Wroclaw Medical University, 50-556 Wroclaw, Poland; martaczwojdzinska@gmail.com; 5 Department of Gynaecological and Obstetrics, District Hospital, 43-200 Pszczyna, Poland; marcinlesniewski@wp.pl; 6 Gynecological and Obstetrician Polyclinic, 15-435 Białystok, Poland; artur_nowak@mp.pl; 7 Clinical Center of Gynecology, Obstetrics and Neonatology, 45-066 Opole, Poland; szynkaruk.malwina@gmail.com

**Keywords:** polycystic ovary syndrome, phenotypes, hormonal status, lipids, disorders

## Abstract

We aimed to evaluate the relationship between selected serum sex hormones and lipid profiles in a group of women with polycystic ovary syndrome (PCOS) dividing according to four phenotypes, value of body mass index (BMI), and presence of hyperlipidemia. The study included 606 Caucasian women. Lipids and selected hormones were estimated using commercially available procedures during hospitalization in 2017. Phenotype of PCOS, BMI value, and hyperlipidemia were significant factors that influenced androgen hormone concentrations, such as total and free testosterone and androstenedione as well as the value of free androgen index (FAI). Moreover, significant changes in concentrations of dehydroepiandrosterone sulphate and sex hormone binding globulin (SHBG) were found between those groups. Higher quartiles of triglyceride concentrations increased the odds ratio of decreased concentrations of SHBG or increased values of FAI, while an adverse relation was found in case of HDL-C. The concentration of estradiol in the blood of women with PCOS was not associated with lipid profile parameters in any investigated groups. Probably, irregularities in sex hormone concentrations during PCOS is not directly associated with lipid profile parameters but could be reflective of the concentration of SHBG or the ratio of SHBG and total testosterone and their association with lipids.

## 1. Introduction

Polycystic ovary syndrome (PCOS) is a common endocrinological disorder in women of reproductive age [1,2,3]. Depending on diagnostic criteria, PCOS affects approximately 6% to 20% of women of reproductive age [4] and is characterized by clinical and/or biochemical hyperandrogenism, ovulatory dysfunction, and polycystic ovarian morphologic features [5]. PCOS predisposes women to various metabolic disorders including dyslipidemia, impaired glucose tolerance, central obesity, and hormonal imbalances [6]. Dyslipidemia and insulin resistance (IR) are some of the most common features observed in women with this syndrome [7]. IR affects approximately 65–70% of women with PCOS, and roughly 70–80% of women with PCOS are overweight or obese [8]. Both IR and accompanying obesity play crucial roles in the pathogenesis of androgen excess in PCOS [9]. Android-type obesity is often accompanied by dyslipidemia with high concentrations of (TG) and low-density lipoproteins (LDL-C), as well as low concentrations of high-density lipoproteins (HDL-C) [10]. Furthermore, abdominal obesity induces ovarian and adrenal androgen production, whereas sex hormone binding globulin (SHBG) level is decreased. In the study conducted by Pasquali et al. it was shown that the increased proportion of small subcutaneous abdominal adipocytes was positively correlated with serum testosterone and androstenedione (AD) levels in women [11]. In addition, it is possible that visceral obesity increases activity in the hypothalamus–pituitary–adrenal axis, which could increase the level of hormones produced by the adrenal cortex adrenal hormones [12].

The drop in SHBG concentration increases testosterone levels, which may further increase abdominal obesity and inflammation, therefore creating a vicious cycle [13]. Moreover, the data acquired in a study of the Chinese population confirmed that overweight or obese women with PCOS had lower serum SHBG concentration when compared to normal weight women with PCOS, and this observation was independent of women’ age [14,15].

In addition, the phenotypic division of patients with PCOS can be associated with different hormonal and metabolic disorders, which in turn may help prognosticate the severity of the patients’ disease [16]. Optimal measures of lipid profile parameters are closely associated with appropriate steroid hormone levels [17]. Concurrently, sex hormones affect many facets of lipid metabolism through interactions with other regulators such as apolipoprotein E and endothelial, hepatic, or lipoprotein lipase [18]. Cholesterol (CHO) is a precursor of five major classes of steroid hormones (adrenal and gonadal hormones), such as testosterone, androstenedione (androgen), estradiol (estrogen), progesterone (progestin), cortisol/corticosterone (glucocorticoid), and aldosterone (mineralocorticoids) [19]. In addition, CHO de novo is synthesized by steroidogenic organs such as ovaries and adrenal glands, which mainly utilize CHO supplied from HDL and LDL, which indicates their important role in maintaining homeostasis in the human body, as well as their participation in hormonal disorders and the development of PCOS [19]. Furthermore, steroid hormones influence the secretion of follicle-stimulating hormone (FSH) and luteinizing hormone (LH) [20]. On the other hand, FSH controls progesterone and estrogen synthesis in ovarian granulosa cells, whereas LH regulates progesterone synthesis in luteinized ovarian granulosa-luteal cells, androgen production in ovarian theca-interstitial cells, and testosterone synthesis in testicular Leydig cells [21]. It was shown that endogenous estrogens and androgens are involved in the metabolism of TG and CHO, and more is known about estrogen than androgen control of lipid metabolism [22]. It was suggested that many metabolic diseases are associated with hormonal disorders [23]. In addition, endocrine and metabolic diseases are among the most frequently occurring contemporary human afflictions [24]. In our study, we aimed to evaluate the relationship between selected serum sex hormones such as FSH, LH, total testosterone (TT), free testosterone (FT), AD, dehydroepiandrosterone sulfate (DHEA-S), estradiol (17-β-E2), 17α-hydroxyprogesterone (17α-OHP), cortisol, SHBG, and lipid profile parameters including CHO, LDL-C, HDL-C, and TG in a group of women with PCOS. Furthermore, we divided patients according to four phenotypes of PCOS, body mass index (BMI), and hyperlipidemia to check how they affect the investigated parameters. We hope that the performed analysis will provide valuable data for clinicians and help clarify the relationships between irregularities in metabolic and hormonal parameters observed in the course of PCOS. 

## 2. Materials and Methods

We conducted a retrospective study on a cohort of 606 women treated for PCOS in the Gynecological Endocrinology Clinic of the Silesian Medical University in Katowice, Poland, in 2017. BMI was calculated using a standard formula: $BMI=\frac{weight\;\left(kg\right)}{height\;\left(m^{2}\right)}$. Normal weight was defined as 18.5–25.0 kg/m^2^, and underweight as BMI < 18.50; overweight as BMI > 25.0 kg/m^2^, and obesity as BMI ≥ 30.0 kg/m^2^. Waist-to-hip ratio (WHR) was assessed as $WHR=\frac{waist\;circumference\;\left(cm\right)}{hip\;circumference\;\left(cm\right)}$. Normal WHR for women was defined as <0.88, and abdominal obesity as ≥0.88. The study was approved by the Bioethical Committee of the Medical University of Silesia. PCOS was diagnosed according to the Rotterdam ESHRE/ASRM criteria from 2003 [25] with at least two of the following three criteria: the existence of oligomenorrhea or amenorrhea defined as less than 2 menstrual cycles in the past 6 months, clinical or biochemical hyperandrogenism, and polycystic appearance of ovary on ultrasonography (USG). In addition, based on the Rotterdam criteria, four different phenotypes of PCOS were recognized according to features: phenotype 1—with clinical and/or biochemical hyperandrogenism, oligomenorrhea, and polycystic ovaries; phenotype 2—with clinical and/or biochemical hyperandrogenism and oligomenorrhea; phenotype 3—with clinical and/or biochemical hyperandrogenism and polycystic ovaries, and phenotype 4—with oligomenorrhea and polycystic ovaries. Patients with other disorders such as type 1 and 2 diabetes (fasting glucose and insulin concentration was assayed as well as oral glucose tolerance test was performed), hypertension (blood pressure was measured), Cushing’s syndrome (the concentration of cortisol at 7:00 a.m., 10:00 p.m., and after 1 mg oral dose of dexamethasone was assayed), late-onset congenital adrenal hyperplasia and adrenal tumor (concentration of 17α-OHP and DHEA-S was investigated), and thyroid dysfunctions (the concentration of thyroid-stimulating hormone (TSH), thyroxine (ft4), triiodothyronine (ft3), and anti-thyroid peroxidase antibody (TPO-Ab) was measured) were excluded from the study. Exposure to tobacco smoke and alcohol abuse were also exclusion criteria. At first, the study involved 787 women, and after excluding some patients according to the criteria described above, the investigation was focused on a group of 606 women. Blood samples were collected during the follicular phase (within 1 and 5 days of the menstrual cycle) in the morning after an overnight fast (>8 h) according to the standard procedures. All laboratory parameters including lipid profile and selected sex hormones were assayed in serum or plasma immediately after blood collection.

### 2.1. Lipid Profile Parameters and Concentration of Glucose and Insulin

The levels of CHO, LDL-C, HDL-C, and TG were estimated by using commercially available test kits (Roche Diagnostics, Indianapolis, IN, USA). Serum levels of cholesterol ≥ 200 mg/dL and triglycerides ≥ 150 mg/dL were considered abnormal [26]. Plasma glucose was determined by using the colorimetric method (Roche Diagnostics, Indianapolis, IN, USA), whereas serum insulin concentration was estimated with ELISA (DRG Instruments GmbH, Marburg, Germany). In addition, each patient underwent a 2-hour oral glucose tolerance test (OGTT) following 75 g of glucose intake.

### 2.2. Hormone Assay

FSH, LH, estradiol (17-β-E2), total testosterone (TT), FT, androstenedione (AD), dehydroepiandrosterone sulfate (DHEA-S), and SHBG were determined by ELISA (DRG Instruments GmbH, Marburg, Germany) with lower limits of sensitivity of 0.86 IU/L, 1.27 IU/L, 9.7 ng/L, 0.083 µg/L, 0.002 ng/L, 0.019 µg/L, 0.044 mg/L, and 0.2 nmol/L, respectively; the respective intra- and inter-assay coefficients of variation were 5.5% and 6.1% for FSH, 5.6% and 6.2% for LH, 4.7% and 7.8% for 17-β-E2, 3.6% and 7.1% for total testosterone, 6.4% and 8.0% for free testosterone, 6.5% and 10.2% for androstenedione, 4.8% and 7.5% for DHEA-S, and 5.3% and 9.0% for SHBG. 17α-hydroxyprogesterone (17α-OHP) and cortisol were assayed by RIA (Diagnostic Products Corporation, Los Angeles, CA, USA) with lower detectable concentrations of 0.2 nmol/L and 5.5 nmol/L, respectively. The respective inter- and intra-assay coefficients of variation were 5.6% and 8.0% for 17-OH-P and 4.3% and 5.2% for cortisol. The ratio of $\frac{LH}{FSH}=\frac{LH\;\left(\frac{IU}{L}\right)}{FSH\;\left(\frac{IU}{L}\right)}$ was calculated. In addition, the FAI value was assessed according to the formula $FAI=\frac{TT\;\left(\frac{nmol}{L}\right)\times 100}{SHBG\;\left(\frac{nmol}{L}\right)}$. 

### 2.3. Statistical Analysis

Statistical calculations were done using the Statistica Software Package, version 13.3 (Polish version; StatSoft, Kraków, Poland). Values were expressed as mean ± SD and median with interquartile range (IQR). The normality of the variables was tested using the Shapiro–Wilk test. The homogeneity of variance was assessed using Levene’s test. The significant difference between two groups was investigated using Student’s t-test (when the normality of distribution and equality of variance were satisfied) or the non-parametric Mann–Whitney U-test (when a lack of normal distribution and variance uniformity were revealed). Differences between four phenotype subgroups and between four subgroups divided according to BMI value were analyzed using Kruskal–Wallis one-way analysis of variance by ranks or one-way analysis of variance (ANOVA) only in cases of HDL-C concentration. Univariate and multiple regression analyses was performed to evaluate the association of hormone concentration with lipid profile parameters. The relationship between HDL-C or TG concentration and abnormal SHBG concentration or FAI value was determined by a binary logistic regression model. In all instances, *p* < 0.05 was considered statistically significant. 

## 3. Results

**Figure 1:**
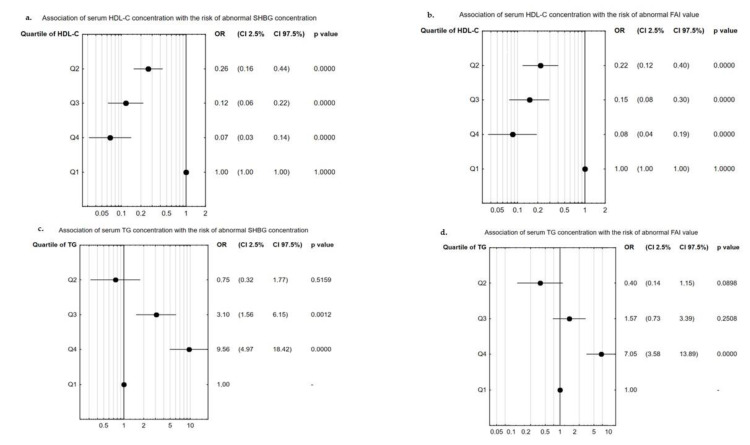
Odds ratio for abnormal serum SHBG concentration or FAI value in quartiles of serum HDL-C or TG level: (**a**,**c**) association between concentration of SHBG and quartiles of HDL-C or TG level; (**b**,**d**) association between value of FAI and quartiles of HDL-C or TG level.

The values obtained in four phenotypes of PCOS are summarized in Table 1. The most common phenotype in our study was type I (*n* = 363/606), followed by type IV (*n* = 97/606), and then II and III (for both *n* = 73/606). We found significant differences in the values of BMI and WHR between women with different phenotypes of PCOS, while the age of study participants was similar in those subgroups. Different phenotypes of PCOS were associated with alterations in concentrations of HDL-C and TG as well as fasting glucose concentration and insulin concentration, both fasting and after OGTT. Moreover, different phenotypes of PCOS were also linked to changes in the concentration of LH, DHEA-S, SHBG, TT, FT, AD, and 17α-OHP and the value of FAI. The highest concentrations of HDL-C and TG were detected in phenotype 1 when compared to phenotypes 2–4 in the case of HDL concentration or to phenotype 3–4 in the case of TG concentration. The highest LH, TT, and 17α-OHP levels were also identified in phenotype 1, while the SHBG concentration was found to be the lowest in said subgroup (Table 1). Similar findings to those observed in phenotype subgroups were demonstrated while analyzing BMI values (Table 2). Significant differences were found in lipid profiles, concentrations of glucose and insulin (both fasting and after OGTT), and the level of steroid hormones, as well as the SHBG concentration. Only LH, FSH, and 17-β-E2 concentration was not significantly associated with BMI value. Higher value of BMI was linked to increased levels of CHO, LDL-C, and TG along with the value of FAI, simultaneously pointing to decreased concentrations of HDL-C and SHBG. The concentration of SHBG in the women with PCOS and BMI > 30.0 was almost 3-fold lower than in the women with BMI<18.5 and 2-fold lower compared to women with healthy BMI value (18.5–25.0) (Table 2). Our results also showed that hyperlipidemia significantly affects steroids hormones such as TT, FT, AD, and cortisol, and FAI value. Additionally, a significant almost 60% drop in SHBG concentration was observed in women with hyperlipidemia. Hyperlipidemia did not change concentrations of LH, FSH, 17-β-E2, 17α-OHP, glucose, or insulin (Table 3). To identify the association between hormone concentration and lipid profiles, firstly univariate regression analysis was performed separately in the whole group of patients and in the patients with identified phenotype 1 of PCOS, the most androgenic type. In both groups, increased concentration of HDL-C was associated with an elevation in SHBG and FSH levels, while a drop in TT or FT concentrations or FAI value was observed. Only in phenotype 1 of PCOS, was the concentration of HDL-C positively correlated with LH concentration and value of the LH/FSH ratio. Furthermore, in phenotype 1, the concentration of HDL-C was positively associated with concentration of cortisol. In the whole group in addition to phenotype 1 of PCOS, higher TG level was linked to increased concentrations of TT and FT and FAI value, and decreased concentration of SHBG. Moreover, in the whole group we also found a positive association between TG level and AD concentration, while in phenotype 1 of PCOS, a negative link between FSH and 17α-OHP concentrations was observed. In the whole group of women with PCOS, fasting glucose concentration was negatively associated with HDL-C concentration, while fasting and after OGTT insulin concentration were positively corelated with TG concentration. In phenotype 1, only concentration of insulin after OGTT was significantly associated with HDL-C concentration. No significant correlations were found between the level of CHO and hormone concentrations in the whole group of women with PCOS and phenotype 1, while LDL-C level was significant correlated only with TT concentration in phenotype 1 (β = 0.13; *p* = 0.041) (Table 4). Using multiple regression analysis, we revealed that the concentration of HDL-C was an independent predictor of increased concentration of SHBG and decreased value of FAI in **the** whole group of women with PCOS and in the phenotype 1 subgroup. A negative correlation was detected between TG concentration and the SHBG concentration or FAI value in all analyzed groups (Table 5). Next, we analyzed the chance of abnormal concentration of SHBG and value of FAI, when we divided women with PCOS according to quartiles of serum HDL-C or TG levels. It was shown that higher concentration of HDL-C (quartile 2–4) was associated with decreased odds ratio of abnormal SHBG concentration incidence. The lowest odds ratio (OR = 1/0.07 = 14.29) of abnormal SHBG was found in the women with the highest concentration of HDL-C (quartile 4); in quartile 3 the odds ratio was about 8.33 (OR = 1/0.12), and in quartile 2 it was 3.85 (OR = 1/0.26). Higher concentrations of TG significantly increased the chance of abnormal SHBG concentration occurring, about 3-fold higher in quartile 3 of TG concentration, and above 9.56-fold higher in quartile 4 (Figure 1a,c). In the case of FAI value, the highest levels of TG (quartile 4) significantly increased the odds ratio of disorder in the FAI value to about 7.07, while an elevation in HDL-C concentration was associated with a decreased odds ratio of a higher value of FAI in quartiles 2 (OR = 1/0.22 = 4.55), 3 (OR = 1/0.15 = 6.67), and 4 (OR = 1/0.06 = 16.67) (Figure 1b,d).

## 4. Discussion

The increasing number of women with PCOS, its significant influence on the quality of life, and the occurrence of other coexisting diseases are growing problems of modern societies and are of constant interest to researchers [27,28]. In fact, diagnosis of diseases associated with hormonal and metabolic disorders has dramatically increased, especially in developed and developing countries. Furthermore, global trends of infertility prevalence in women are constantly increasing, and recently, PCOS has been identified as the leading cause of anovulatory infertility [29]. In women with PCOS, endocrine and metabolic disorders are commonly observed [30]. Contemporary medicine links the changes in lipid profiles with many disorders, including mental health [31,32], which could help explain multiple components of PCOS. Moreover, there is a large difference between full-blown PCOS—phenotype 1, which is associated with a higher risk of metabolic disorders and cardiovascular diseases—and phenotype 4, which is the least severe phenotype [16]. Therefore we aimed to investigate the relationship between selected hormone status and lipid profiles in a large group of Polish women with PCOS (*n* = 606) divided according to four phenotypes; we also analyzed those relationships in the patients with phenotype 1. A study conducted by Carmina et al. [33] focused on the metabolic alterations in various phenotypes of PCOS in a large group of Sicilian women with a low prevalence of obesity, and it was shown that phenotype 2 was the most metabolically affected phenotype, followed by phenotype 1. In our study, the highest metabolic disorder was detected in phenotype 1. The differentiations between those women with PCOS are probably associated with lifestyle of women. Similar to our study, the women with phenotype 4 were characterized by the lowest metabolic disorders. Hormonal disorders can be caused by lower concentrations of SHBG. We found that a drop in SHBG concentration in the serum of women with PCOS was dependent on the four phenotypes of PCOS and BMI value. Moreover, the lowest concentration of SHBG was observed in phenotype 1, when compared to other phenotypes. When we assessed the influence of BMI value on SGBH concentration, we found an almost 2-fold lower concentration in obese women (BMI > 30.0) than in women with normal weight (BMI between 18.5 and 25.0). The drop in SHBG concentration was mainly associated with decreased level of HDL-C and increased level of TG, which was shown by a linear regression analysis, not only in the whole group but also in phenotype 1 of PCOS. We also found that a higher odds ratio of abnormal concentration of SHBG was associated with the lower quartile of HDL-C level. Similar results were obtained by Gunning and Fauser [34] in a large cohort study, who also observed decreased concentrations of SHBG and HDL-C in women with PCOS and claimed that lower concentrations of those parameters as well as increased levels of CHO, LDL-C, and TG in PCOS increased risk of cardiovascular diseases. Earlier meta-analysis performed by de Groot [35] showed that increased BMI is not the sole cause of the increased cardiovascular risk in women with PCOS. In addition, in our study in the whole group of women with PCOS as well as in patients with phenotype 1 of PCOS, we found a significant correlation between HDL-C (negative) or TG (positive) level and TT or FT concentration. A recent study of Hestiantoro et al. [36] also suggested that TG is the main determinant of FT level in PCOS and hypothesized that phenotype 1 is influenced by both hormonal and metabolic dysfunctions. Furthermore, the authors also observed that SHBG was negatively correlated with FT [36]. The review provided by Qu and Donnelly [37] also suggested a close association between the SHBG concentration and changes in lipids and indicated that serum SHBG levels may be a useful diagnostic biomarker and therapeutic target for managing women with PCOS, though more clinical studies are needed to assess the usefulness of SHBG as a biomarker for identifying young women who may later develop PCOS. In our study we confirmed that SHBG concentration was significantly decreased depending on phenotypes of PCOS, BMI value, or lipid disorders. Moreover, a decreased concentration of SHBG was in direct relation to a lower concentration of HDL-C. Similar results were earlier obtained in Chinese women with PCOS, in whom decreased concentrations of SHBG were closely associated with decreased levels of HDL-C, independent of IR or obesity [38]. An opposite correlation was revealed between concentration of TG and SHBG in the whole group and in phenotype 1 women. An increased level of TG was in inverse proportion to SHBG concentration, which also suggests a close relationship between those metabolic and hormonal parameters. Moreover, an increased odds ratio of abnormal concentration of SHBG was associated with a higher quartile of TG level. Recent research of Zhu et al. [15] showed that in PCOS, patients with both hyperandrogenism and hyperinsulinemia had reduced serum SHBG level, and that a decreased level of SHBG promoted lipid disorders. In our study, we revealed, using multiple regression analysis, that concentration of SHBG was significantly associated with changes in HDL-C and TG concentration, but further investigation is needed to explain which mechanism is involved in those relationships. It was also claimed that only unbound testosterone is active and able to bind the androgen receptor of target tissues of the body. Contemporary medicine has been questioning the free hormone hypothesis because testosterone can also be weakly bound to albumin; therefore, both FT and weakly bound testosterone contribute to androgen effects. In our study, women who had the lowest concentration of SHBG were characterized by higher concentrations of both TT and FT, which confirmed that a lower concentration of SHBG was associated not only with higher concentration of FT, but also TT [39]. In addition, using univariate regression analysis in both groups, we revealed that TT and FT concentrations were negatively related to HDL-C level and positively to TG. 

FAI value is considered to be another sensitive parameter that can accurately reflect the bioactive androgen levels [9], and the value of FAI is in an inverse proportion to the concentration of SHBG [39]. In our study, we found that the value of BMI or presence of hyperlipidemia affected this parameter. Similar to the concentration of SHBG, obese women had 3-fold higher FAI values than women with normal weight. Moreover, multiple regression analysis showed that FAI value was significantly associated with HDL-C level (negatively) and with TG (positively) in the whole group of women with PCOS and in phenotype 1 patients, which confirmed results obtained earlier by other authors, who argued that FAI value should be considered the best predictive factor throughout life of women having PCOS [40]. Moreover, higher quartiles of TG concentration increased the odds ratio of decreased concentrations of SHBG or increased values of FAI, which also suggests that abnormal TG levels can be associated with hormonal imbalances.

Another crucial steroid hormone produced by the adrenal cortices is DHEA-S [41], which together with dehydroepiandrosterone (DHEA) is the most abundant circulating steroid hormone in the human body [42]. The association between DHEA-S concentration and metabolic disturbances during PCOS is controversial [43]. A study conducted by Paschou et al. [43] revealed that the concentration of DHEA-S was not associated with significant alteration in lipids profile parameters, despite the higher concentrations of TT and AD, whereas a study conducted by Meirow et al. [44] showed that the concentration of DHEA-S was positively correlated with the level of CHO, LDL-C, and TG, and negatively with HDL-C in the blood of patients with IR. In our study we found that both phenotypes of PCOS and BMI value significantly modulated the concentration of DHEA-S. Moreover, similarly to results obtained by Paschou et al., we did not observe any significant association between DHEA-S concentration and lipid profile parameters.

In the current literature, there is little information about the relationship between estradiol, progesterone or androstenedione, and lipid profiles in young women with PCOS; therefore, in our study, we also evaluated the relationship between lipid profiles and those hormones. Many publications are focused on menopausal or postmenopausal women [45,46]. The results obtained in the menopausal women clearly demonstrated that reduced estradiol concentration could be associated with lipid disorders [45], which increase risk of cardiovascular diseases. Unfortunately, in our study we did not find any significant differences in estradiol concentration between women with different phenotypes, BMI values, or lipids level. No significant association between the level of estradiol and lipids profile was found either. Neither phenotype of PCOS, BMI value, or disorders in lipid profiles changed the concentration of estradiol, which could be associated with young age of women. In case of progesterone, we found that the phenotype of PCOS or value of BMI could be significant factors influencing the concentration of 17α-OHP. In addition, only in phenotype 1 of PCOS, the concentration of 17α-OHP was weakly but significantly inversely linked to TG concentration. A similar weak correlation was found between AD concentration and HDL-C (negative) or TG (positive) levels in the whole group. An earlier study by Blumenfeld et al. [47] demonstrated that PCOS is associated with dysregulation in glucocorticoid degradation. In general, population cortisol excretion positively correlated with the value of BMI and WHR and negatively with HDL-C concentration [48]. Similarly, in our study we observed that BMI value was linked to changes in cortisol concentration. When we evaluated the association between lipid profiles and concentrations of cortisol, we only found that higher concentrations of cortisol were in direct proportion to HDL-C concentration in the phenotype 1 of PCOS. 

In our study, the women with phenotype 1 of PCOS had the highest median value of BMI (BMI = 24.0 (21.0–30.1)) than other phenotypes. The median value of BMI in four phenotypes was rather low, which could be associated with young age of patients. The study conducted by Bagir et al. [49] showed higher mean values of BMI (for phenotype 1—27.9 ± 7.5, for phenotype 2—26.1 ± 5.3, for phenotype 3—24.3 ± 4.2, and for phenotype 4—27.9 ± 5.2), even if the mean age was similar to that in our study. Our results also suggest that the relationship between high BMI and individual phenotypic features of the Rotterdam criteria that characterize PCOS remains unclear, as was claimed by Neubronner et al. [50]. They also did not observe any effect of BMI on rates of oligomenorrhea or on the ovarian parameters in PCOS women, which could explain the lack of the association between BMI value and phenotypes of PCOS. This part of the study should be continued. 

LH and FSH are not steroids hormones, but they induce their synthesis [51]. Higher levels of LH induce androgen synthesis in ovarian theca cells. In turn, hyperandrogenemia decreases levels of both estradiol and progesterone in gonadotropic hypothalamic cells, reinforcing gonadotropin-releasing hormone and LH hypersecretion [52]. Studies conducted by other authors revealed a weak but significant positive correlation between FSH and CHO (β = 0.13) or HDL-C (β = 0.13) in post-menopausal women [53]. Similarly, in our present study, we also observed in all examined groups a positive association between FSH and HDL-C concentration with the highest coefficient correlation in the women with hyperlipidemia (β = 0.34). In addition, in phenotype 1 of PCOS, we also detected an inverse correlation between FSH and TG concentration. 

## 5. Conclusions

1. It seems that sex hormone concentrations are mainly indirectly associated with lipids.
2. Changes in concentration of SHBG and value of FAI were significantly associated with disturbance in HDL-C and TG levels.
3. Higher quartiles of TG concentration increased the odds ratio of decreased concentration of SHBG or increased value of FAI, while higher quartiles of HDL-C level decreased disturbances in SHBG concentration and FAI value.
4. The phenotypes of PCOS, BMI value, and hyperlipidemia are significant factors that influence androgen hormone concentrations in women with PCOS.
5. The concentration of estradiol in the blood of women with PCOS was not associated with lipid profile parameters.

## Figures and Tables

**Table 1:** Investigated parameters in the subgroups divided according to phenotypes of PCOS.

Parameters	Phenotype 1 *n* = 363	Phenotype 2 *n* = 73	Phenotype 3 *n* = 73	Phenotype 4 *n* = 97	*p* Value
Age (years)	24.0 (22.0–28.0)	27.0 (23.0–33.0)	25.0 (22.0–28.5)	25.0 (22.0–29.0)	>0.05
BMI (kg/m2)	24.0 (21.0–30.1)	22.8 (19.6–27.1)	22.4 (19.8–26.5)	22.9 (20.4–27.2)	0.016
WHR	0.8 (0.8–0.9)	0.8 (0.8–0.9)	0.8 (0.8–0.9)	0.8 (0.8–0.9)	0.017
CHO (mg/dL)	177.0 (160.0–197.0)	176.0 (161.0–201.0)	167.5 (153.0–194.0)	179.0 (158.0–197.0)	>0.05
HDL-C (mg/dL)	51.2 ± 12.2	56.5 ± 11.9	57.4 ± 10.2	55.7 ± 11.3	0.001
LDL-C (mg/dL)	104.0 (90.0–122.0)	104.0 (84.0–118.0)	95.0 (80.0–110.0)	104.5 (90.5–119.0)	>0.05
TG (mg/dL)	97.0 (68.0–154.0)	81.5 (60.0–119.0)	78.0 (65.0–96.0)	82.0 (61.5–111.5)	0.001
Glucose (mg/dl)	89.9 (85.0; 93.0)	91.0 (84.5; 94.5)	87.0 (84.0; 90.0)	88.0 (84.5; 91.5)	>0.05
Insulin (mU/ml)	8.0 (5.5; 12.8)	8.8 (5.2; 12.5)	7.0 (5.6; 9.3)	6.3 (4.9; 8.6)	0.002
Glucose after OGTT	107.0 (89.0; 128.0)	98.0 (83.0; 116.0)	93.5 (80.0; 112.0)	94.0 (85.0; 110.0)	0.007
Insulin after OGTT	45.2 (27.4; 83.7)	34.4 (22.5; 64.4)	29.3 (19.0; 49.9)	28.8 (20.9; 42.9)	0.000
LH (lU/L)	7.9 (5.5–11.0)	6.0 (3.6–7.5) c	5.6 (4.7–6.7)	6.8 (4.7–8.6)	0.045
FSH (lU/L)	5.8 (4.9–6.6)	6.0 (4.9–7.2)	6.4 (5.2–7.4)	5.9 (5.0–7.3)	>0.05
LH/FSH	1.3 (1.0–1.9)	0.9 (0.6–1.4)	0.8 (0.7–1.1)	1.1 (0.8–1.4)	>0.05
DHEA-S (µg/mL)	310.9 (234.9–402.0)	297.3 (230.5–383.1)	336.4 (206.1–429.5)	259.2 (192.9–310.9)	0.010
SHBG (nmol/L)	47.5 (29.0–67.7)	59.3 (31.2–90.0)	69.2 (47.1–87.7)	56.5 (42.9–82.5)	0.001
TT (ng/mL)	0.4 (0.3–0.6)	0.3 (0.2–0.5)	0.3 (0.2–0.4)	0.3 (0.2–0.3)	0.001
FT (pg/mL)	1.9 (1.2–2.7) f	1.3 (1.0–2.0)	1.4 (0.9–2.3)	1.0 (0.7–1.5)	0.001
FAI	0.9 (0.5–1.5)	0.5 (0.3–0.9)	0.4 (0.2–0.9)	0.4 (0.2–0.8)	0.011
AD (ng/mL)	3.6 (2.6–4.6)	3.0 (2.3–4.0)	2.8 (2.2–3.6)	2.3 (1.8–2.8)	0.001
17-β-E2 (pg/mL)	33.5 (22.9–47.2)	35.2 (26.6–49.2)	32.1 (23.8–39.1)	29.9 (18.2–37.9)	0.157
17α-OHP (nmol/L)	1.8 (1.4–2.2)	1.5 (1.2–1.9)	1.5 (1.3–1.9)	1.4 (1.2–1.8)	0.003
Cortisol (µg/dL)	13.6 (11.1–16.1)	13.7 (11.3–16.4)	13.3 (10.2–14.8)	13.2 (10.7–15.1)	0.359

Values are shown as median with IQR or mean ± SD. Legend: PCOS—polycystic ovary syndrome; BMI—body mass index; WHR—waist-to-hip ratio; CHO—cholesterol; HDL-C—high density lipoprotein; LDL-C—low density lipoprotein; TG—triglycerides; LH—luteinizing hormone; FSH—follicle-stimulating hormone; DHEA-S—dehydroepiandrosterone sulfate; SHBG—sex hormone-binding globulin; TT—total testosterone; FT—free testosterone; FAI—free androgen index; AD—androstenedione; 17-β-E2—17 β-estradiol;17-OH P—17-α-hydroxyprogesterone.

**Table 2:** Investigated parameters in the subgroups divided according to the value of BMI.

Parameters	BMI				
	<18.5*n* = 43	18.5–25.0 *n* = 336	25.0–30.0 *n* = 98	>30.0 *n* = 129	*p* Value
Age (years)	25.0 (21.0–31.0)	25.0 (22.0–29.0)	25.5 (23.0–31.0)	27.0 (23.0–32.0)	>0.05
WHR	0.8 (0.7–0.8) d/e/f	0.8 (0.8–0.9)	0.9 (0.8–0.9)	0.9 (0.9–1.0)	0.001
CHO (mg/dL)	170.0 (156.0–189.0)	174.0 (156.0–194.0	179.0 (162.0–200.5)	189.0 (169.0–209.0)	0.003
HDL-C (mg/dL)	62.0 ± 13.1	58.4 ± 11.0	49.8 ± 11.3	45.2 ± 9.4	0.001
LDL-C (mg/dL)	93.0 (83.0–106.0)	102.0 (86.0–116.0)	107.0 (92.0–122.0)	114.0 (93.5–131.0)	0.001
TG (mg/dL)	65.5 (56.0–85.0)	73.0 (60.0–93.0)	105.0 (80.0–162.0)	148.0 (105.0–200.0)	0.001
Glucose (mg/dL)	86.0 (81.0; 90.0)	88.0 (84.0; 91.0)	91.0 (87.0; 95.0)	92.0 (88.0; 97.0)	0.001
Insulin (mU/mL)	4.8 (3.3; 6.1)	6.0 (4.2; 8.2)	8.3 (7.0; 12.1)	13.8 (10.0; 18.9)	0.001
Glucose after OGTT	93.5 (79.0; 112.0)	93.5 (82.0; 109.0)	109.0 (92.0; 130.0)	115.5 (97.5; 137.0)	0.001
Insulin after OGTT	27.4 (17.9; 45.0)	28.8 (20.0; 45.1)	45.3 (28.6; 70.6)	45.3 (28.6; 70.6)	0.001
LH (lU/L)	6.8 (3.4–8.7)	6.6 (4.7–10.4)	6.4 (4.4–9.4)	6.8 (3.4–8.7)	>0.05
FSH (lU/L)	6.2 (4.6–8.7)	6.1 (5.1–7.2)	5.8 (4.8–6.9)	6.0 (5.0–6.7)	>0.05
LH/FSH	0.8 (0.5–1.4)	1.1 (0.7–1.6)	1.1 (0.7–1.6)	1.0 (0.7–1.4)	>0.05
DHEA-S (µg/mL)	255.7 (180.5–315.6)	282.8 (212.3–367.5)	330.2 (254.3–439.9)	305.0 (204.9–397.3)	0.001
SHBG (nmol/L)	90.7 (69.1–123.7)	66.3 (50.2–87.3)	40.8 (29.9–57.0)	30.0 (20.4–44.3)	0.001
TT (ng/mL)	0.2 (0.2–0.4)	0.3 (0.2–0.4)	0.4 (0.3–0.5)	0.4 (0.3–0.5)	0.001
FT (pg/mL)	1.1 (0.7–1.7)	1.3 (0.8–2.0)	1.8 (1.1–2.6)	1.8 (1.1–2.7)	0.001
FAI	0.3 (0.2–0.5)	0.4 (0.2–0.8)	0.9 (0.5–1.5)	1.2 (0.7–2.0)	0.001
AD (ng/mL)	2.5 (1.7–3.4)	2.7 (1.9–3.7)	3.4 (2.6–4.8)	3.0 (2.1–3.9)	0.001
17-β-E2 (pg/mL)	34.1 (19.9–48.0)	33.0 (22.9–46.1)	31.1 (19.9–45.0)	32.8 (25.5–43.8)	>0.05
17α-OHP (nmol/L)	1.5 (1.0–1.9)	1.6 (1.2–2.0)	1.9 (1.5–2.3)	1.6 (1.3–2.0)	0.003
Cortisol (µg/dL)	13.6 (11.1–16.1)	14.0 (11.7–16.2)	12.7 (10.8–15.8)	13.3 (10.7–15.8)	0.026

Values are shown as median with IQR or mean ± SD. Legend: PCOS—polycystic ovary syndrome; BMI—body mass index; WHR—waist-to-hip ratio; CHO—cholesterol; HDL-C—high density lipoprotein; LDL-C—low density lipoprotein; TG—triglycerides; LH—luteinizing hormone; FSH—follicle-stimulating hormone; DHEA-S—dehydroepiandrosterone sulfate; SHBG—sex hormone-binding globulin; TT—total testosterone; FT—free testosterone; FAI—free androgen index; AD—androstenedione; 17-β-E2—17 β-estradiol;17-OH P—17-α-hydroxyprogesterone.

**Table 3:** Investigated parameters in the subgroups divided according to hyperlipidemia (CHO > 200.00 mg/dL and TG > 150 mg/dL).

Parameters	Lipid Profile		
	Normal Value *n* = 557	Hyperlipidemia *n* = 49	*p* Value
Age (years)	25.0 (22.0–30.0)	28.0 (24.0–34.0)	<0.013
BMI (kg/m2)	22.5 (20.1–27.2)	31.1 (25.5–35.2)	<0.001
WHR	0.8 (0.8–0.9)	0.9 (0.9–1.0)	<0.001
Glucose (mg/dL)	89.0 (85.0–93.0)	91.0 (88.0–95.0)	>0.05
Insulin (mU/mL)	6.7 (4.8–10.7)	12.9 (8.1–15.7)	>0.05
Glucose after OGTT	100.0 (85.0–117.0)	121.0 (98.0–148.0)	>0.05
Insulin after OGTT	35.1 (21.9–57.5)	79.1 (39.8–114.0)	>0.05
LH (lU/L)	6.4 (4.4–9.5)	6.6 (4.1–10.2)	>0.05
FSH (lU/L)	6.1 (5.0–7.2)	5.7 (5.1–6.8)	>0.05
LH/FSH	1.0 (0.7–1.5)	1.1 (0.8–1.5)	>0.05
DHEA-S (µg/mL)	291.6 (211.2–376.2)	297.5 (221.4–352.3)	>0.05
SHBG (nmol/L)	57.1 (36.5–82.1)	38.5 (23.7–52.6)	<0.001
TT (ng/mL)	0.3 (0.2–0.5)	0.4 (0.2–0.6)	0.045
FT (pg/mL)	1.4 (0.9–2.2)	2.0 (0.8–3.0)	0.003
FAI	0.6 (0.3–1.1)	1.2 (0.7–1.7)	0.018
AD (ng/mL)	2.8 (1.9–3.8)	3.7 (2.7–4.7)	0.009
17-β-E2 (pg/mL)	33.0 (23.8–45.8)	32.5 (21.3–49.1)	>0.05
17α-OHP (nmol/L)	1.6 (1.2–2.0)	1.6 (1.2–2.0)	>0.05
Cortisol (µg/dL)	13.6 (11.5–16.1)	14.8 (12.3–17.4)	0.022

Values are shown as median with IQR. Legend: PCOS—polycystic ovary syndrome; BMI—body mass index; WHR—waist-to-hip ratio; CHO—cholesterol; HDL-C—high density lipoprotein; LDL-C—low density lipoprotein; TG—triglycerides; LH—luteinizing hormone; FSH—follicle-stimulating hormone; DHEA-S—dehydroepiandrosterone sulfate; SHBG—sex hormone-binding globulin; TT—total testosterone; FT—free testosterone; FAI—free androgen index; AD—androstenedione; 17-β-E2—17 β-estradiol;17-OH P—17-α-hydroxyprogesterone.

**Table 4:** Univariate regression analysis in the whole group of women with PCOS and in the subgroup with phenotype 1.

Covariate	HDL-C (mg/dL)	TG (mg/dL)
Whole group of women with PCOS		
Glucose (mg/dL)	β = −0.36; *p* = 0.014	NS
Insulin (mU/mL)	NS	β = 0.49; *p* < 0.001
Insulin after OGTT	NS	β = 0.27; *p* = 0.046
FSH (lU/L)	β = 0.09; *p* = 0.032	NS
SHBG (nmol/L)	β = 0.33; *p* < 0.001	β = −0.29; *p* < 0.001
TT (ng/mL)	β = −0.20; *p* < 0.001	β = 0.17; *p* < 0.001
FT (pg/mL)	β = −0.18; *p* < 0.001	β = 0.19; *p* < 0.001
FAI	β = −0.37; *p* < 0.001	β = 0.38; *p* < 0.001
AD (ng/mL)	β = −0.10; *p* = 0.015	β = 0.10; *p* = 0.018
	Phenotype 1 of PCOS	
Insulin after OGTT	β = −0.30; *p* < 0.001	NS
LH (lU/L)	β = 0.19; *p* = 0.002	NS
FSH (lU/L)	β = 0.16; *p* = 0.011	β = −0.17; *p* = 0.011
LH/FSH	β = 0.15; *p* = 0.002	NS
SHBG (nmol/L)	β = 0.26; *p* < 0.001	β = −0.26; *p* < 0.001
TT (ng/mL)	β = −0.15; *p* = 0.016	β = 0.18; *p* = 0.004
FT (pg/mL)	β = −0.13; *p* = 0.047	β = 0.19; *p* = 0.003
FAI	β = −0.38; *p* < 0.001	β = 0.39; *p* < 0.001
17α-OHP (nmol/L)	NS	β = −0.16; *p* = 0.010
Cortisol (µg/dL)	β = 0.19; *p* = 0.003	NS

Legend: PCOS—polycystic ovary syndrome; CHO—cholesterol; HDL-C—high density lipoprotein; LDL-C—low density lipoprotein; TG—triglycerides; LH—luteinizing hormone; FSH—follicle-stimulating hormone; DHEA-S—dehydroepiandrosterone sulfate; SHBG—sex hormone-binding globulin; TT—total testosterone; FT—free testosterone; FAI—free androgen index; AD—androstenedione; 17-β-E2—17 β-estradiol; 17-OH P—17-α-hydroxyprogesterone; NS—non-significant.

**Table 5:** Multiple regression analysis in the whole group of women with PCOS and in the phenotype 1.

Whole Group of PCOS		β Value	Summarized Multiple Regression Analysis
SHBG (nmol/L)	HDL-C (mg/dL)	β = 0.25; *p* < 0.001	R = 0.37; R^2^ = 0.14; Adjusted R^2^ = 0.13; F = 46.5; *p* < 0.001
	TG (mg/dL)	β = −0.18; *p* < 0.001	
FAI	HDL-C (mg/dL)	β = −0.25; *p* < 0.001	R = 0.44; R^2^ = 0.20; Adjusted R^2^ = 0.20; F = 71.26; *p* < 0.001
	TG (mg/dL)	β = 0.28; *p* < 0.001	
**Phenotype 1 of PCOS**		**β Value**	**Summarized Multiple Regression Analysis**
SHBG (nmol/L)	HDL-C (mg/dL)	β = 0.18; *p* = 0.010	R = 0.30; R^2^ = 0.09; Adjusted R^2^ = 0.08; F = 12.19; *p* < 0.001
	TG (mg/dL)	β = −0.18; *p* = 0.010	
FAI	HDL-C (mg/dL)	β = −0.24; *p* < 0.001	R = 0.45; R^2^ = 0.20; Adjusted R^2^ = 0.19; F = 30.88; *p* < 0.001
	TG (mg/dL)	β = 0.28; *p* < 0.001	

Legend: PCOS—polycystic ovary syndrome; CHO—cholesterol; HDL-C—high density lipoprotein; LDL-C—low density lipoprotein; TG—triglycerides; SHBG—sex hormone-binding globulin; FAI—free androgen index; NS—non-significant.

## Data Availability

The data presented in this study are available upon request from the corresponding author.
